# Genetic variability of inflammation and oxidative stress genes does not play a major role in the occurrence of adverse events of dopaminergic treatment in Parkinson’s disease

**DOI:** 10.1186/s12974-019-1439-y

**Published:** 2019-02-27

**Authors:** Sara Redenšek, Dušan Flisar, Maja Kojović, Milica Gregorič Kramberger, Dejan Georgiev, Zvezdan Pirtošek, Maja Trošt, Vita Dolžan

**Affiliations:** 1 Pharmacogenetics Laboratory, Institute of Biochemistry, Faculty of Medicine, University of Ljubljana, Vrazov trg 2, 1000 Ljubljana, Slovenia; 2 Department of Neurology, University Medical Centre Ljubljana, Zaloška cesta 2, 1000 Ljubljana, Slovenia

**Keywords:** Parkinson’s disease, Susceptibility, Polymorphism, Inflammation, Oxidative stress, Adverse events

## Abstract

**Background:**

Inflammation and oxidative stress are recognized as important contributors to Parkinson’s disease pathogenesis. As such, genetic variability in these pathways could have a role in susceptibility for the disease as well as in the treatment outcome. Dopaminergic treatment is effective in management of motor symptoms, but poses a risk for motor and non-motor adverse events. Our aim was to evaluate the impact of selected single-nucleotide polymorphisms in genes involved in inflammation and oxidative stress on Parkinson’s disease susceptibility and the occurrence of adverse events of dopaminergic treatment.

**Methods:**

In total, 224 patients were enrolled, and their demographic and clinical data on the disease course were collected. Furthermore, a control group of 146 healthy Slovenian blood donors were included for Parkinson’s disease’ risk evaluation. Peripheral blood was obtained for DNA isolation. Genotyping was performed for *NLRP3* rs35829419, *CARD8* rs2043211, *IL1β* rs16944, *IL1β* rs1143623, *IL6* rs1800795, *CAT* rs1001179, *CAT* rs10836235, *SOD2* rs4880, *NOS1* rs2293054, *NOS1* rs2682826, *TNF-α* rs1800629, and *GPX1* rs1050450. Logistic regression was used for analysis of possible associations.

**Results:**

We observed a nominally significant association of the *IL1β* rs1143623 C allele with the risk for Parkinson’s disease **(**OR = 0.59; 95%CI = 0.38–0.92, *p* = 0.021). *CAT* rs1001179 A allele was significantly associated with peripheral edema (OR = 0.32; 95%CI = 0.15–0.68; *p* = 0.003). Other associations observed were only nominally significant after adjustments: *NOS1* rs2682826 A allele and excessive daytime sleepiness and sleep attacks (OR = 1.75; 95%CI = 1.00–3.06, *p* = 0.048), *SOD2* rs4880 T allele and nausea/vomiting (OR = 0.49, 95%CI = 0.25–0.94; *p* = 0.031), *IL1β* rs1143623 C allele and orthostatic hypotension (OR = 0.57, 95%CI = 0.32–1.00, *p* = 0.050), and *NOS1* rs2682826 A allele and impulse control disorders (OR = 2.59; 95%CI = 1.09–6.19; *p* = 0.032). We did not find any associations between selected polymorphisms and motor adverse events.

**Conclusions:**

Apart from some nominally significant associations, one significant association between *CAT* genetic variability and peripheral edema was observed as well. Therefore, the results of our study suggest some links between genetic variability in inflammation- and oxidative stress-related pathways and non-motor adverse events of dopaminergic treatment. However, the investigated polymorphisms do not play a major role in the occurrence of the disease and the adverse events of dopaminergic treatment.

**Electronic supplementary material:**

The online version of this article (10.1186/s12974-019-1439-y) contains supplementary material, which is available to authorized users.

## Background

Inflammation and oxidative stress are recognized as important mechanisms in pathogenesis of Parkinson’s disease’ (PD) [1]. However, it is still unknown whether these interconnected and self-propagating pathways are causative for PD or do they occur in response to the death of dopaminergic neurons in substantia nigra pars compacta (SNpc) and other brain regions affected in PD [2]. Furthermore, genetic factors, such as single-nucleotide polymorphisms (SNPs) were shown to modify these pathways, but their impact on the risk of PD and on the outcome of PD treatment has not been studied yet [3].

Neuroinflammation is one of the main pathological hallmarks of PD [4]. Microglia as the key innate immune system cells of the brain play the key role in this process [5]. They trigger inflammation by release of proinflammatory cytokines (IL-1β, TNF-α, IL-6), reactive oxygen and nitrogen species (ROS and RNS, respectively), chemokines, and complement proteins [2, 6, 7]. ROS and aggregated α-synuclein may act as damage-associated molecular patterns and may thus activate NLRP3 inflammasome, which is essential for IL-1β activation [8]. Pathway analysis of genome wide association studies data revealed the role of genetic variability in inflammation pathway in PD [9]. Moreover, pronounced microglia activation was detected by positron emission tomography (PET) imaging in various brain regions in PD [10–12]. Furthermore, levels of IL-1β, TNF-α, and IL-6, among other cytokines, have been shown to be elevated in peripheral blood and cerebrospinal fluid of PD patients [13–19].

Three main processes lead to ROS production in PD brain [20]. First, a source of ROS in the dopaminergic neurons is dopamine metabolism itself. Dopamine is oxidized by either monoamine oxidase or auto-oxidation, leading to production of hydrogen peroxide or superoxide radicals, respectively [21]. Second, complex I deficiency contributes to mitochondrial dysfunction, which also leads to major ROS production [20, 22]. Third source of ROS is chronic neuroinflammation [23]. Activated microglia contributes to oxidative burden by producing elevated amounts of inducible nitric oxide synthase (NOS), which adds to high levels of nitric oxide produced by neuronal NOS [6, 24]. ROS-related potential PD biomarkers were already detected in blood, urine, and cerebrospinal fluid [7, 25]. Main ROS scavenging enzymes are superoxide dismutase (SOD2), catalase (CAT), and glutathione peroxidase (GPX1). Their activity, which is influenced by the genetic variability, is usually elevated in PD [26, 27]. Oxidative stress in PD was detected also by brain PET imaging [28].

Several studies evaluating the effect of genetic polymorphisms or alterations in proteins’ functions in the inflammation- and oxidative stress-related genes on the risk for PD in either cell culture, animal models, or human samples have already been performed, but conflicting results were presented. Some studies have already linked the NLRP3 function to PD risk [29, 30]. Furthermore, *IL-1β* rs16944 has been associated with PD risk [31, 32]. Also *TNF-α* rs1800629 has been associated with the disease risk [31], but later studies did not confirm the result [32, 33]. Moreover, two other *TNF-α* SNPs [34, 35] and *IL6* rs1800795 [36] showed the same association. Bridge between inflammatory processes and ROS/RNS stress may be presented by *NOS1*. *NOS1* rs2682826 has already been associated with levodopa-induced dyskinesia [24]. *NOS1* polymorphisms have also been connected to the disease risk [37–39], although results are discrepant [40]. GPX1 [41–43] and CAT [44] activities might be related to PD pathogenesis. *SOD2* rs4880 was not found to be associated with PD risk [45, 46].

Activated inflammation pathways and increased oxidative stress contribute to death of dopaminergic neurons in SNpc and consequential dopamine depletion. Therefore, dopamine replacement strategy has a central role in PD management [47]. Dopaminergic therapy is effective for the treatment of motor symptoms of PD, but poses a risk for the development of motor and non-motor adverse events (AEs). Motor fluctuations and dyskinesia are the most frequent motor AEs. Although they primarily reflect severity of the disease process and mostly occur after long-term levodopa administration, they are sometimes observed shortly after treatment initiation. Non-motor AEs, such as excessive daytime sleepiness (EDS) and sleep attacks, visual hallucinations (VH), nausea/vomiting, orthostatic hypotension (OH), peripheral edema (PE), and impulse control disorders (ICDs), are associated with both levodopa and dopamine agonists (DAs) [48, 49].

Although the association of genetic variability of inflammation and oxidative stress genes with the risk of PD has already been explored to some extent, the association with the treatment outcome has not been studied yet. First, our aim was to evaluate the possible association between selected SNPs in inflammation and oxidative stress pathways with the risk of PD. Furthermore, it has been suggested that pathways involved in disease pathogenesis may also influence treatment outcome [3]. The latter being said, our aim was also to investigate the association of selected SNPs in the above mentioned pathways with motor and non-motor AEs of dopaminergic treatment in PD.

## Materials and methods

### Study participants

A total of 224 PD patients were enrolled in this retrospective cohort study. Patients were recruited in succession and evaluated at the Department of Neurology, University Medical Centre Ljubljana, Slovenia between October 2016 and January 2018. Inclusion criteria were (1) diagnosis of PD according to the UK Parkinson Disease Society Brain Bank criteria [50] by an experienced movement disorders specialist, (2) appropriate clinical data available, (3) at least 3 months of levodopa and/or dopamine agonists treatment duration, and (4) ongoing dopaminergic therapy with levodopa and/or dopamine agonists. Patients with atypical and secondary forms of parkinsonism were not included in the study.

Patients and their caregivers underwent a structured interview to obtain clinical and demographic data. Information was additionally obtained from medical records. We focused on eight AEs of dopaminergic treatment as main end points: motor fluctuations, dyskinesia, EDS and sleep attacks, VH, nausea/vomiting, OH, PE, and ICDs. The AE was defined as absent or present according to clinical examination, clinical documentation, and patients’ answers to specific questions. The time of the AE occurrence after treatment initiation was not taken into account.

A control group of 146 healthy unrelated Slovenian blood donors, aged 50 to 65 years, were included in the study for the purpose of PD susceptibility evaluation.

The study protocol was approved by the Slovenian Ethics Committee for Research in Medicine (KME 42/05/16). All subjects gave written informed consent in accordance with the Declaration of Helsinki.

### SNP selection

Nine candidate genes were studied on the basis of their direct involvement in the signaling cascades of the inflammation and oxidative stress pathways [26, 51, 52], which are both involved in PD pathogenesis: four functional SNPs *NLRP3* rs35829419, *CARD8* rs2043211, *GPX1* rs1050450, and *SOD2* rs4880; five promoter SNPs *IL1β* rs16944, *IL1β* rs1143623, *TNF-α* rs1800629, *IL6* rs1800795, and *CAT* rs1001179*.* Additionally, the SNP function prediction tool was used [53] to select two SNPs in *NOS1* (rs2293054 and rs2682826) and one SNP in *CAT* (rs10836235). We selected SNPs with minor allele frequency at least 2% and with determined function based on literature and/or in silico prediction.

### DNA isolation and genotyping

Peripheral blood samples were obtained for DNA extraction. Genomic DNA was isolated using the FlexiGene DNA Kit (Qiagen, Hilden, Germany) according to the manufacturer’s protocol. Genotyping was performed for 12 SNPs. Nine of them were genotyped with KASPar assays (KBiosciences, Herts, UK and LGC Genomics, UK) according to the manufacturer’s instructions. Three of the SNPs were genotyped with TaqMan genotyping assays (Applied Biosystems, Foster City, CA, USA) also according to the manufacturer’s protocol. Ten percent of samples were genotyped in duplicate as quality control, and all the results were concordant.

### Statistical analysis

Median and 25th to 75th percentile range were used to describe central tendency and variability of continuous variables, while frequencies were used to describe the distribution of categorical variables. The agreement of genotype frequencies with Hardy-Weinberg equilibrium and univariate analyses of the individual effects of categorical variables on the AEs were conducted by chi-squared test. Nonparametric Mann-Whitney *U* test was used for the assessment of the effect of numerical data on the AEs. Logistic regression was used to calculate odds ratios (ORs), and 95% confidence intervals (CIs) to examine the associations of selected SNPs and clinical data with the risk for AEs of dopaminergic treatment. Dominant, additive, and recessive genetic models were used for analysis depending on the genotype frequencies. All statistical tests were two-sided. Bonferroni correction was used to account for multiple comparisons to prevent false positive results. For genetic data, *p* values up to 0.0042 (0.05/12) were considered statistically significant, while *p* values between 0.0042 and 0.0500 were considered nominally significant. For clinical data, *p* values up to 0.0063 (0.05/8) were considered statistically significant, while *p* values between 0.0063 and 0.0500 were considered nominally significant. The study power was calculated for each of the eight AEs separately due to their different frequencies. Three allele frequencies were used for power calculations for each AE, namely minimum polymorphic allele frequency of 6%, average polymorphic allele frequency of 30%, and maximum polymorphic allele frequency of 69%. Power calculations were conducted by the PS Power and sample size calculations, version 3.0, and are presented in the Additional file 1: Table S1. All statistical analyses were carried out by IBM SPSS Statistics, version 21.0 (IBM Corporation, Armonk, NY, USA).

## Results

We evaluated the possible associations of tested SNPs with PD susceptibility. The control group consisted of 146 healthy blood donors with the median age of 56 years. There were 113 men and 33 women in the control group.

General demographic and clinical characteristics of PD patients are presented in Table 1. Median age of patients at enrolment was 72.5 years (65.6–78.0), and median dopaminergic therapy duration was 7.3 years (3.6–13.5). The list and frequency of AEs are also presented in Table 1. In total, 194 (86.6%) patients experienced at least one of the AEs. Patients who experienced any AE had earlier disease onset, longer disease duration and levodopa treatment duration, and higher levodopa equivalent dose (LED) (calculated according to [54]) and have already been treated with DAs (all *p* < 0.001).

**Table 1:** Demographic and clinical data of PD patients with the list of AEs

Characteristic		All patients (*N* = 224)
Sex	Female (%)	95 (42.4)
	Male (%)	129 (57.6)
Side of first symptoms	Left (%)	88 (39.3)
	Both (%)	20 (8.9)
	Right (%)	116 (51.8)
Tremor-predominant PD	No (%)	41 (18.3)
	Yes (%)	183 (81.7)
Ever being treated with DAs^c^	No (%)	55 (25.0)
	Yes (%)	165 (75.0)
Age at diagnosis	Median (25–75%), years	62.2 (55.0–71.6)
Disease duration	Median (25–75%), years	7.6 (3.8–14.0)
Levodopa treatment duration^b^	Median (25–75%), years	6.1 (2.3–11.0)
LED at enrolment^c, d^	Median (25–75%), mg/day	970 (600–1343.63)
Adverse event	Number (%) of patients experiencing the adverse event	
Motor fluctuations	119 (53.1)	
Dyskinesia	98 (43.8)	
EDS and sleep attacks	79 (35.5)	
Visual hallucinations^a^	57 (25.6)	
Nausea/vomiting^a^	66 (29.6)	
Orthostatic hypotension^a^	84 (37.7)	
Peripheral edema^a^	44 (19.7)	
Impulse control disorders^a^	32 (14.3)	

^a^Data missing for one patient

^b^Data missing for three patients

^c^Data missing for four patients

^d^LED calculated according to [54]

Frequencies for the 12 investigated SNPs in both the patient and the control group are presented in the Table 2. The genotype distributions did not deviate from the Hardy-Weinberg equilibrium (HWE) except for *NLRP3* rs35829419 and *NOS1* rs2293054 in the control group where the frequencies did not meet the HWE requirements. Moreover, they also deviated from the genotype frequencies reported for the European population within the 1000 Genomes project. Consequently, these two SNPs were excluded from the PD susceptibility analysis.

**Table 2:** Genotype frequencies of the study and control group with the risk analysis’ results

Gene	Genotype	*N* (%) in the study group	*N* (%) in the control group	OR (95%CI)	*p* value	OR** (95%CI**)	*p* value**
*NLRP3* rs35829419	CC	196 (87.5)	120 (82.2)				
	CA	27 (12.1)	22 (15.1)				
	AA	1 (0.4)	4 (2.7)				
*CARD8* rs2043211	AA	105 (46.9)	66 (45.2)	Ref.		Ref.	
	AT	89 (39.7)	64 (43.8)	0.87 (0.56–1.36)	0.553	0.85 (0.53–1.38)	0.515
	TT	30 (13.4)	16 (11.0)	1.18 (0.60–2.33)	0.636	1.48 (0.72–3.04)	0.290
	AT+TT	119 (53.1)	80 (54.8)	0.94 (0.62–1.42)	0.753	0.97 (0.62–1.52)	0.893
IL1β* rs16944	AA	25 (11.2)	21 (14.4)	0.64 (0.33–1.24)	0.183	0.76 (0.37–1.55)	0.447
	AG	89 (39.7)	66 (45.2)	0.72 (0.46–1.13)	0.157	0.72 (0.45–1.16)	0.176
	GG	110 (49.1)	59 (40.4)	Ref.		Ref.	
	AA+AG	114 (50.9)	87 (59.6)	0.70 (0.46–1.07)	0.101	0.73 (0.46–1.14)	0.166
*IL1β* rs1143623	GG	131 (58.5)	67 (45.9)	Ref.		Ref.	
	GC	79 (35.3)	64 (43.8)	**0.63 (0.41–0.98)**	**0.041**	**0.62 (0.38–0.99)**	**0.043**
	CC	14 (6.3)	15 (10.3)	0.48 (0.22–1.05)	0.065	0.47 (0.20–1.11)	0.084
	GC+CC	93 (41.6)	79 (54.1)	**0.60 (0.40–0.92)**	**0.018**	**0.59 (0.38–0.92)**	**0.021**
*TNF-α* rs1800629	GG	156 (69.6)	99 (67.8)	Ref.		Ref.	
	GA	59 (26.3)	42 (28.8)	0.89 (0.56–1.43)	0.631	0.88 (0.53–1.46)	0.629
	AA	9 (4.0)	5 (3.4)	1.14 (0.37–3.51)	0.816	1.51 (0.47–4.83)	0.487
	GA+AA	68 (30.3)	47 (32.2)	0.92 (0.59–1.44)	0.709	0.95 (0.59–1.53)	0.824
*IL6* rs1800795	GG	65 (29.0)	45 (30.8)	Ref.		Ref.	
	GC	120 (53.6)	67 (45.9)	1.24 (0.77–2.01)	0.383	1.04 (0.62–1.74)	0.885
	CC	39 (17.4)	34 (23.3)	0.79 (0.44–1.44)	0.449	0.72 (0.38–1.36)	0.312
	GC+CC	159 (71.0)	101 (69.2)	1.09 (0.69–1.72)	0.711	0.93 (0.57–1.52)	0.782
*NOS1* rs2293054	GG	118 (52.7)	87 (59.6)				
	GA	89 (48.2)	43 (29.5)				
	AA	17 (7.6)	16 (11.0)				
*NOS1* rs2682826	GG	108 (48.2)	73 (50.0)	Ref.		Ref.	
	GA	101 (45.1)	57 (39.0)	1.20 (0.77–1.86)	0.422	1.16 (0.72–1.85)	0.548
	AA	15 (6.7)	16 (11.0)	0.63 (0.30–1.36)	0.242	0.64 (0.28–1.44)	0.279
	GA+AA	116 (51.8)	73 (50.0)	1.07 (0.71–1.63)	0.737	1.04 (0.67–1.63)	0.857
*GPX1* rs1050450	CC	115 (51.3)	71 (49.0)	Ref.		Ref.	
	CT	92 (41.1)	60 (41.4)	0.95 (0.61–1.47)	0.807	0.97 (0.61–1.56)	0.908
	TT	17 (7.6)	14 (9.7)	0.75 (0.35–1.61)	0.461	0.69 (0.30–1.56)	0.371
	CT+TT	109 (48.7)	74 (51.1)	0.92 (0.61–1.40)	0.703	0.93 (0.59–1.45)	0.738
*CAT* rs10836235	CC	172 (76.8)	117 (80.1)	Ref.		Ref.	
	CT	47 (21.0)	25 (17.1)	1.28 (0.75–2.19)	0.371	1.34 (0.76–2.38)	0.311
	TT	5 (2.2)	4 (2.7)	0.85 (0.22–3.23)	0.812	0.65 (0.15–2.72)	0.552
	CT+TT	52 (23.2)	29 (19.8)	1.22 (0.73–2.03)	0.446	1.24 (0.72–2.13)	0.438
*CAT* rs1001179	GG	122 (54.5)	89 (61.4)	Ref.		Ref.	
	GA	92 (41.1)	51 (35.2)	1.32 (0.85–2.04)	0.219	1.01 (0.63–1.62)	0.974
	AA	10 (4.5)	5 (3.4)	1.46 (0.48–4.42)	0.504	1.77 (0.54–5.79)	0.348
	GA+AA	102 (45.6)	56 (38.6)	1.34 (0.88–2.06)	0.173	1.08 (0.68–1.70)	0.757
*SOD2* rs4880	CC	65 (29.0)	40 (27.4)	Ref.		Ref.	
	CT	108 (48.2)	69 (47.3)	0.96 (0.59–1.58)	0.882	0.95 (0.56–1.62)	0.861
	TT	51 (22.8)	37 (25.3)	0.85 (0.48–1.51)	0.577	0.74 (0.39–1.37)	0.335
	CT+TT	159 (71.0)	106 (72.6)	0.92 (0.58–1.47)	0.735	0.88 (0.53–1.45)	0.605

Alleles are provided as constructed by the manufacturer. The ancestral allele is matched with the dbSNP

*Recessive model was used

**Adjusted for sex and age

Nominally significant results are written in bold text

When PD susceptibility analysis was performed, univariate logistic regression analysis showed no statistically significant results. The only nominally significant result was the association between *IL1β* rs1143623 and the risk for PD. The carriers of at least one C allele and also heterozygotes had lower odds for developing PD. The association remained nominally significant even after adjustment for age and sex (OR = 0.59; 95%CI = 0.38–0.92, *p* = 0.021). The results are presented in the Table 2.

Univariate logistic regression analysis of the effect of categorical clinical data (sex, side of first symptoms, tremor-predominant PD, and ever being treated with DAs) on the occurrence of AEs (Table 3) showed that female patients had more than three times greater odds for the development of nausea/vomiting (OR = 3.22; 95%CI = 1.77–5.85, *p* = < 0.001). Furthermore, patients ever being treated with DAs also had more than three times higher odds for developing nausea/vomiting (OR = 3.19; 95%CI = 1.41–7.19, *p* = 0.005) and 12 times higher odds for developing ICDs (OR = 12.00; 95%CI = 1.60–90.21, *p* = 0.016). Regarding motor AEs, tremor-predominant PD decreased odds for the development of motor fluctuations and dyskinesia for more than twice (OR = 0.40; 95%CI = 0.19–0.83, *p* = 0.014 and OR = 0.33; 95%CI = 0.16–0.67, *p* = 0.002, respectively), while ever being treated with DAs increased odds for the development of these two AEs for more than six and five times, respectively (OR = 6.82; 95%CI = 3.28–14.19, *p* = < 0.001 and OR = 5.43; 95%CI = 2.50–11.81, *p* = < 0.001, respectively). Also continuous clinical data (age at diagnosis, disease duration, levodopa treatment duration, and LED at enrolment) showed some significant associations with certain AEs (Table 3), but all of the ORs appeared to be close to one, which indicates a rather small clinical effect.

**Table 3:** Univariate analysis of the influence of clinical data on the occurrence of AEs

		EDS and sleep attacks		Visual hallucinations		Nausea and vomiting		Orthostatic hypotension	
		OR (95% CI)	*p* value	OR (95% CI)	*p* value	OR (95% CI)	*p* value	OR (95%CI)	*p* value
Sex (male = ref.)		0.75 (0.43–1.32)	0.322	0.74 (0.40–1.38)	0.347	**3.22 (1.77–5.85)**	**< 0.001**	0.706 (0.41–1.23)	0.218
Side of first symptoms (left = ref.)	Both	0.83 (0.29–2.38)	0.726	0.35 (0.08–1.63)	0.181	1.06 (0.37–3.08)	0.911	1.72 (0.65–4.57)	0.278
	Right	1.14 (0.64–2.03)	0.660	1.30 (0.69–2.46)	0.412	1.07 (0.58–1.97)	0.824	0.98 (0.55–1.74)	0.933
Tremor-predominant PD (No = ref.)		0.82 (0.41–1.65)	0.578	0.92 (0.43–1.99)	0.837	0.89 (0.43–1.84)	0.743	0.57 (0.29–1.13)	0.107
Ever being treated with DAs (No = ref.)		1.81 (0.91–3.58)	0.089	1.79 (0.84–3.85)	0.134	**3.19 (1.41–7.19)**	**0.005**	0.74 (0.40–1.37)	0.337
Age at diagnosis		1.00 (0.98–1.02)	0.871	**0.97 (0.94–0.99)**	**0.006**	**0.97 (0.95–1.00)**	**0.026**	1.01 (0.99–1.04)	0.281
Disease duration		1.04 (1.00–1.08)	0.066	**1.13 (1.08–1.19)**	**< 0.001**	1.02 (0.98–1.06)	0.366	1.04 (1.00–1.08)	0.080
Levodopa treatment duration		1.04 (0.99–1.08)	0.112	**1.15 (1.09–1.21)**	**< 0.001**	1.00 (0.96–1.05)	0.940	**1.07 (1.02–1.12)**	**0.005**
LED at enrolment		1.00 (1.00–1.00)	0.106	**1.00 (1.00–1.00)**	**0.004**	1.00 (1.00–1.00)	0.869	1.00 (1.00–1.00)	0.085
		Peripheral edema		Impulse control disorders		Motor fluctuations		Dyskinesia	
Sex (male = ref.)		0.66 (0.33–1.31)	0.229	0.58 (0.26–1.29)	0.181	0.97 (0.57–1.64)	0.899	1.03 (0.61–1.76)	0.905
Side of first symptoms (left = ref.)	Both	2.44 (0.79–7.50)	0.120	0.33 (0.04–2.69)	0.300	0.37 (0.13–1.06)	0.065	0.59 (0.21–1.68)	0.324
	Right	1.56 (0.75–3.27)	0.234	1.22 (0.56–2.68)	0.613	1.15 (0.66–2.01)	0.620	1.24 (0.71–2.17)	0.446
Tremor-predominant PD (No = ref.)		1.02 (0.43–2.39)	0.969	0.97 (0.37–2.54)	0.954	**0.40 (0.19–0.83)**	**0.014**	**0.33 (0.16–0.67)**	**0.002**
Ever being treated with DAs (No = ref.)		2.44 (0.97–6.14)	0.058	**12.00 (1.60–90.21)**	**0.016**	**6.82 (3.28–14.19)**	**< 0.001**	**5.43 (2.50–11.81)**	**< 0.001**
Age at diagnosis		1.00 (0.97–1.03)	0.977	**0.93 (0.90–0.96)**	**< 0.001**	**0.89 (0.86–0.92)**	**< 0.001**	**0.88 (0.85–0.91)**	**< 0.001**
Disease duration		1.010 (0.96–1.06)	0.682	1.04 (0.99–1.0)	0.114	**1.44 (1.31–1.58)**	**< 0.001**	**1.33 (1.24–1.44)**	**< 0.001**
Levodopa treatment duration		1.03 (0.98–1.08)	0.310	1.01 (0.95–1.07)	0.872	**1.42 (1.29–1.56)**	**< 0.001**	**1.32 (1.22–1.42)**	**< 0.001**
LED at enrolment		1.00 (1.00–1.00)	0.212	1.00 (1.00–1.00)	0.140	**1.01 (1.00–1.01)**	**< 0.001**	**1.00 (1.00–1.00)**	**< 0.001**

Significant and nominally significant results are written in bold text

Univariate analysis of the effect of genetic variability on the occurrence of AEs also showed some significant and nominally significant associations. Significantly lower odds for the PE occurrence were detected in the polymorphic *CAT* rs1001179 A allele carriers (OR = 0.32; 95%CI = 0.15–0.68, *p* = 0.003). Furthermore, heterozygotes had significantly lower odds for PE development (OR = 0.33; 95%CI = 0.15–0.70, *p* = 0.004) as well. Carriers of the *NOS1* rs2682826 A allele had nominally significant higher odds for developing EDS and sleep attacks (OR = 1.75; 95%CI = 1.00–3.06, *p* = 0.048). Under additive genetic model, only carriers of the AA genotype had nominally significant higher odds for this AE development (OR = 3.73; 95%CI = 1.22–11.35, *p* = 0.021). Heterozygotes for the *GPX1* rs1050450 had nominally significant higher odds for VH development (OR = 2.01; 95%CI = 1.07–3.77, *p* = 0.030). Nausea and vomiting were also less likely to occur in carriers of the *SOD2* rs4880 T allele (OR = 0.51; 95%CI = 0.28–0.94, *p* = 0.030), but the association was only nominally significant. The *IL1β* rs1143623 T allele showed a nonsignificant trend towards association with lower odds for developing OH (OR = 0.57; 95%CI = 0.32–1.00, *p* = 0.050), while heterozygotes had nominally significant lower odds for developing OH (OR = 0.51; 95%CI = 0.28–0.93, *p* = 0.028). The investigated SNPs were not associated with ICD development or with the motor AEs after univariate analysis. Results of the univariate analyses under dominant and recessive models for genetic data are presented in Table 4. Results of the univariate analysis under additive genetic model are presented in Additional file 1: Tables S3–S5.

**Table 4:** Univariate analysis of the influence of genetic polymorphisms on the occurrence of AEs

SNP	EDS and sleep attacks		Visual hallucinations		Nausea and vomiting		Orthostatic hypotension	
	OR (95% CI)	*p* value	OR (95% CI)	*p* value	OR (95% CI)	*p* value	OR (95% CI)	*p* value
*NLRP3* rs35829419	0.70 (0.30–1.68)	0.430	0.77 (0.30–2.00)	0.593	0.77 (0.31–1.91)	0.570	0.76 (0.33–1.76)	0.520
*CARD8* rs2043211	0.856 (0.50–1.48)	0.581	0.80 (0.44–1.45)	0.457	1.27 (0.71–2.27)	0.414	1.01(0.59–1.74)	0.961
IL1β* rs16944	1.25 (0.72–2.16)	0.435	1.31 (0.72–2.40)	0.380	0.72 (0.41–1.29)	0.273	0.69 (0.40–1.18)	0.173
*IL1β* rs1143623	1.19 (0.69–2.08)	0.532	1.13 (0.61–2.07)	0.702	0.73 (0.40–1.32)	0.295	0.57 (0.32–1.00)	0.050
*TNF-α* rs1800629	0.83 (0.45–1.52)	0.547	0.78 (0.40–1.54)	0.477	0.83 (0.44–1.57)	0.559	0.98 (0.54–1.77)	0.943
*IL6* rs1800795	0.90 (0.50–1.65)	0.740	0.67 (0.35–1.27)	0.218	1.23 (0.64–2.36)	0.529	1.34 (0.73–2.48)	0.343
*NOS1* rs2293054	1.33 (0.77–2.30)	0.312	1.02 (0.56–1.86)	0.960	0.84 (0.50–1.49)	0.542	0.82 (0.48–1.42)	0.480
*NOS1* rs2682826	**1.75 (1.00–3.06)**	**0.048**	1.25 (0.68–2.29)	0.471	0.58 (0.32–1.03)	0.064	1.02 (0.60–1.76)	0.933
*GPX1* rs1050450	1.13 (0.65–1.96)	0.663	1.79 (0.97–3.31)	0.061	0.63 (0.35–1.13)	0.124	0.92 (0.54–1.59)	0.770
*CAT* rs10836235	1.08 (0.56–2.05)	0.827	1.65 (0.84–3.26)	0.150	1.58 (0.82–3.05)	0.174	1.84 (0.98–3.47)	0.059
*CAT* rs1001179	1.00 (0.58–1.74)	0.994	0.75 (0.41–1.37)	0.345	0.76 (0.42–1.36)	0.348	0.90 (0.52–1.55)	0.693
*SOD2* rs4880	1.20 (0.65–2.22)	0.554	0.96 (0.50–1.85)	0.896	**0.51 (0.28–0.94)**	**0.030**	1.26 (0.69–2.31)	0.450
SNP	Peripheral edema		Impulse control disorders		Motor fluctuations		Dyskinesia	
*NLRP3* rs35829419	0.87 (0.31–2.43)	0.790	0.69 (0.20–2.42)	0.559	0.74 (0.33–1.63)	0.449	0.96 (0.43–2.14)	0.919
*CARD8* rs2043211	0.60 (0.31–1.17)	0.133	0.55 (0.26–1.17)	0.122	0.92 (0.54–1.55)	0.744	1.33 (0.78–2.23)	0.288
IL1β* rs16944	0.60 (0.31-1.17)	0.133	1.72 (0.80–3.71)	0.167	1.11 (0.66–1.87)	0.700	1.09 (0.64–1.84)	0.762
*IL1β* rs1143623	0.59 (0.29–1.19)	0.140	1.10 (0.52–2.35)	0.800	1.13 (0.66–1.92)	0.665	1.28 (0.75–2.19)	0.366
*TNF-α* rs1800629	0.85 (0.41–1.76)	0.655	1.74 (0.80–3.76)	0.162	1.08 (0.61–1.91)	0.799	1.11 (0.63–1.97)	0.714
*IL6* rs1800795	0.95 (0.46–1.96)	0.890	0.87 (0.39–1.95)	0.731	0.62 (0.34–1.11)	0.108	0.80 (0.45–1.43)	0.447
*NOS1* rs2293054	1.45 (0.75–2.82)	0.270	0.99 (0.47–2.10)	0.979	1.05 (0.62–1.78)	0.854	0.73 (0.43–1.24)	0.238
*NOS1* rs2682826	1.43 (0.73–2.79)	0.296	1.93 (0.88–4.22)	0.100	0.89 (0.53–1.51)	0.663	0.82 (0.48–1.39)	0.459
*GPX1* rs1050450	0.75 (0.39–1.46)	0.400	0.79 (0.37–1.67)	0.531	1.55 (0.92–2.63)	0.103	1.18 (0.70–2.01)	0.533
*CAT* rs10836235	0.70 (0.30–1.63)	0.410	1.39 (0.60–3.23)	0.446	1.15 (0.62–2.14)	0.663	1.39 (0.75–2.59)	0.301
*CAT* rs1001179	**0.32 (0.15–0.68)**	**0.003**	0.67 (0.31–1.45)	0.314	0.92 (0.54–1.55)	0.750	1.28 (0.75–2.18)	0.362
*SOD2* rs4880	0.75 (0.37–1.52)	0.422	0.89 (0.40–2.00)	0.777	1.25 (0.70–2.22)	0.456	1.04 (0.58–1.86)	0.897

Significant and nominally significant results are written in bold text. Homozygotes for wild-type alleles were used as reference, except for one SNP

*Recessive model was used

Adjustments of statistically significant and nominally significant genetic associations for clinical data were carried out in the multivariate logistic regression analysis. Since all four continuous clinical parameters (age at diagnosis, disease duration, levodopa treatment duration, LED at enrolment) appeared to be mutually correlated, which was checked by the Spearman’s correlation coefficient, only age at diagnosis was used for adjustments. Additionally, significant and nominally significant genetic associations (see Table 4) were also adjusted for statistically significant and nominally significant categorical clinical data (see Table 3).

Associations of the *NOS1* rs2682826 with EDS and sleep attacks, *IL1β* rs1143623 with OH, and *CAT* rs1001179 with PE, were adjusted only for age at diagnosis. *CAT* rs1001179 A allele carriers had statistically significant lower odds for developing PE even after adjustment (OR = 0.32; 95%CI = 0.15–0.68, *p* = 0.003). The association of *NOS1* rs2682826 A allele with higher odds for developing EDS and sleep attacks remained nominally significant (OR = 1.75; 95%CI = 1.00–3.06, *p* = 0.048). Furthermore, *IL1β* rs1143623 C allele retained a nonsignificant trend towards association with lower odds for developing OH (OR = 0.57; 95%CI = 0.32–0.99, *p* = 0.050). Furthermore, the *SOD2* rs4880 T allele association with nausea/vomiting was adjusted for sex, ever being treated with DAs, and age at diagnosis. The genetic association was still nominally significantly associated with lower odds for developing nausea/vomiting even after the adjustment (OR = 0.49; 95%CI = 0.25–0.94, *p* = 0.031). Results are presented in Table 5.

**Table 5:** Results of the multivariate logistic regression

Association		OR adj.^a^	95% CI	*p* value
SNP	Adverse event			
*NOS1* rs2682826	EDS and sleep attacks	1.75	1.00–3.06	**0.048**
*SOD2* rs4880	Nausea/vomiting	0.49	0.25–0.94	**0.031**
*IL1β* rs1143623	Orthostatic hypotension	0.57	0.32–0.99	0.050
*CAT* rs1001179	Peripheral edema	0.32	0.15–0.68	**0.003**
*NOS1* rs2682826	Impulse control disorders	2.59	1.09–6.19	**0.032**

^a^Homozygotes for wild-type allele were used as reference. Adjustments are stated in the text

Significant and nominally significant results are written in bold text

Additionally, after a thorough inspection of nonsignificant results of univariate analyses of genetic data and review of the literature, another nominally significant association was found after adjustment for significant clinical parameters. *NOS1* rs2682826 A allele carriers had higher odds for developing ICDs after adjusting for age at diagnosis and ever being treated with DAs (OR = 2.59; 95%CI = 1.09–6.19, *p* = 0.032) (Table 5).

## Discussion

In this study, we evaluated the effect of selected SNPs from inflammation and oxidative stress pathways on the risk for PD and AEs occurrence due to dopaminergic treatment of PD. Several studies evaluating the effect of genetic polymorphisms or alterations in proteins’ functions in the inflammation- and oxidative stress-related pathways on the PD risk and pathogenesis have already been performed [31, 32, 34–39]. However, no studies evaluating genetic variability in these pathways affecting the occurrence of AEs of dopaminergic treatment have been conducted to date. The main finding of our study is that evaluated selected SNPs from pathways affecting the disease pathogenesis might influence the occurrence of non-motor AEs, but according to these results do not affect the occurrence of motor AEs. We found a strong association between *CAT* rs1001179 A allele and the occurrence of PE.

We found no statistically significant associations between tested SNPs and the PD susceptibility, although it is known that genetic defects in inflammation and oxidative stress pathways are involved in PD pathogenesis [3, 51, 55]. Only *IL1β* rs1143623 showed a nominally significant association between carriers of at least one C allele and lower odds for PD occurrence. Our inability to detect any significant associations may be due to a rather small control group. Furthermore, the control and study groups were not matched by sex and age.

EDS and sleep attacks affected 35.5% of patients in our cohort, which is in agreement with previous data stating that this AE manifests in up to 50% of patients [56, 57]. We observed a trend, although not significant, that EDS and sleep attacks are correlated with disease duration, the dose of medication, and with DAs’ administration, which is consistent with the literature [56, 58, 59]. It has been debated several times whether EDS and sleep attacks are a matter of disease pathology or dopaminergic treatment. A clear connection to dopaminergic treatment, especially DAs, has been confirmed in several studies [56, 60]. Furthermore, we detected that the occurrence of this AE might also harbor a genetic component related to oxidative stress pathway as carriers of the *NOS1* rs2682826 A allele had almost two times greater odds for developing this AE in our cohort. The hypothesis that *NOS1* genotype might affect sleep cycle has already been investigated. It was shown that *NOS1* knockout mice spent less time in rapid eye movement (REM) sleep phase and non-REM sleep cycle during the night, which could lead to EDS and sleep attacks during the day [61, 62]. As rs2682826 presumably influences miRNA binding [53], it could lower *NOS1* gene expression and consequently cause daytime wakefulness disturbances by the mechanism similar to the above mentioned mouse model.

In total, 29.6% of patients in our cohort experienced nausea/vomiting, and according to the literature, this is the most common AE of dopaminergic replacement therapy [63]. We detected a trend towards association of the AE with sex, DAs, and age at diagnosis. The association of this AE with DAs’ administration has been thoroughly studied before [57, 63]. Our data suggest that *SOD2* rs4880 may play a role in this AE’s occurrence. Carriers of the *SOD2* rs4880 T allele had nominally significant lower odds for developing nausea/vomiting. This SNP has been reported to decrease enzyme’s function [64]. There is currently no data to support the hypothesis of involvement of oxidative stress in nausea/vomiting. But according to various studies, PD pathogenesis starts in the gut and slowly progresses towards brainstem via the vagus nerve [65]. Aggregates of α-synuclein have been found in the enteric nervous system (ENS) [65]. ENS is responsible for the regulation of many gastrointestinal (GI) functions, including motility and fluid secretion. When dysfunctional, different GI problems can occur, including nausea/vomiting [66]. It has been shown in diabetes that oxidative stress in the ENS might cause GI complications [67]. As aggregated α-synuclein increases oxidative stress [68], the latter could also be the case in PD. Therefore, genetic variability, such as *SOD2* rs4880 might play a role in the nausea/vomiting development as also suggested by our study. Although nausea/vomiting may also result from the direct effect of levodopa on the area postrema of the brain stem [69], there is no experimental data supporting the involvement of oxidative stress in these AEs via this central pathway.

Another common AE of dopaminergic treatment is OH. It affected 37.7% of patients in our cohort, which is comparable to the data in literature [70]. However, as PD patients are inherently prone to autonomic dysfunction, it is hard to distinguish between OH as a symptom and as an AE [63, 71]. Our data suggest that this AE is significantly associated with levodopa treatment duration, but clinical relevance of this result is not conclusive due to OR being close to one. Our results also indicate an association of OH with *IL1β* rs1143623 C allele, which decreases the promoter activity [72]. Dopaminergic drugs lower blood pressure through vasodilatation and decrease in catecholamine release. The hypotensive effect of levodopa usually abates, but the hypotensive effect of DAs persists. The combination of different dopaminergic drugs poses the highest risk for this AE [63, 71]. Some studies explored the connection between inflammation and OH [73, 74], but they focused more on classical OH rather than on neurogenic OH, typical for PD. Nevertheless, our study was the first to point out a possible association between OH in PD patients and inflammation pathways in connection to dopaminergic treatment. This warrants further research to find new pathways involved in OH in PD, which could be relevant for the blood lowering effect of dopaminergic drugs.

Rates of peripheral edema vary from 5 to 16% of patients treated with DAs [63], which is slightly less compared to our data as 19.7% of patients in our cohort were affected with edema. Furthermore, we observed a tendency that patients ever being treated with DAs are more than twice as likely to develop this AE compared to patients never being treated with DAs, which is in concordance with the published literature [57]. Our data also suggest that *CAT* rs1001179 A allele significantly lowers odds for developing the edema for more than two times. This SNP has been reported to increase gene’s expression [75]. The mechanism of this AE is still not fully understood. Presented results suggest that oxidative stress and further antioxidant defense are involved in the occurrence of this AE. According to the previous reports, PE might also occur due to the peripheral effects of dopamine [63].

Furthermore, ICDs appeared to be significantly associated with DAs and with age at diagnosis. DAs have already been strongly associated with this AE [57], which is also evident in our results as DAs increased odds for developing the AE 12 times compared to patients never being treated with DAs. The association was nominally significant. According to our results, patients that were diagnosed older had lower odds for the AE development; however, as OR appeared to be close to one, the association’s clinical relevance is questionable. Results of our study also showed a nominally significant association of the *NOS1* rs2682826 A allele with higher odds for ICDs after adjustment for significant clinical parameters. SNPs of *NOS1* were already associated with several psychiatric disorders such as obsessive compulsive disorder, anxiety, and depression [76, 77].

Our study did not confirm the results of Santos-Lobato et al., which showed an association of the *NOS1* rs2682826 with levodopa-induced dyskinesia [24]. We also did not find associations of the AEs with other studied genes, even though some of them showed associations with PD risk in previous reports [31, 32, 36]. Our study showed that patients treated with DAs had higher odds for developing motor AEs, which was not in concordance with everyday clinical practice and expectations. The result could be explained by the fact that patients, who were already treated with DAs, also had higher LED at enrolment and longer disease duration, which are all risk factors for motor AEs.

Although our study presents novel findings and is designed with a different pathway-based approach to clarifying AEs’ mechanisms, some limitations have to be considered. The control group in the risk analysis is not matched by sex and age. However, we have considered this in the statistical analysis by adjusting for these two parameters. The patient cohort is of moderate size, although it is comparable to the sample sizes of similar PD pharmacogenetics studies. The time of AEs’ occurrence in relation to medication initiation was not taken into account. All of the AEs were analyzed as categorical variables, but with the use of clinical scales to evaluate the severity of various AEs, we could look into possible associations in more depth. Furthermore, prospective study would have a greater chance to detect even subtler and timely relations between treatment and AEs. It should also be noted that our results should be validated in a different independent patient population, before they could be applied in a clinical practice.

Nevertheless, even though it is known that AEs of dopaminergic therapy can be alleviated to some extent by appropriate treatment modifications in many PD patients, AEs still impact their quality of life [78]. In light of this, mechanisms of the AEs’ development must be determined, so predictive biomarkers can be established in order to prevent or minimize their occurrence and to be especially cautious with patients at higher risk to timely take appropriate measures. Patients could be stratified into groups with detectable deficits in inflammation or oxidative stress pathways, so supplementary therapy could be more specific, e.g., anti-inflammatory therapy for patients with deficits in inflammation pathways or antioxidants for patients with inadequate antioxidant defense. In this way, new knowledge on PD genetics could help us guide the treatment [79]. We included genes and SNPs with broad implications in PD and other inflammation-associated brain conditions in the reported study. This type of studies on PD and similar diseases will hopefully someday enable construction of inflammation and/or oxidative stress pathway gene panels. These panels would serve for testing patients with different but related diseases to personalize their treatment.

To the best of our knowledge, this is the first study using pathway-based approach to address the relationship between inflammation and oxidative stress polymorphisms and risk for PD or AEs of dopaminergic treatment. We were able to detect some indication of possible associations of genetic variability in *IL1β* with the risk for PD or *NOS1*, *SOD2*, *IL1β*, and *CAT* with certain non-motor AEs of dopaminergic replacement therapy; however, the evidence presented here is limited. Further association and functional studies are warranted.

## Conclusions

The results of this study confirm the possible association of the inflammation pathway with the risk for PD. Furthermore, the results of this study are the first indication that inflammation and oxidative stress pathways may be involved in the pathogenesis of non-motor AEs of dopaminergic treatment in PD, although they may not play a major role in the process. Further studies on independent samples are warranted to confirm the involvement of genetic variability in these pathways on the occurrence of AEs of dopaminergic treatment in PD. Furthermore, functional studies would possibly lead us to new knowledge on AEs’ pathogenesis and their possible management. For now, SNPs of *NOS1*, *SOD2*, *IL1β*, and *CAT* present possible candidates for future studies on predictive biomarkers of non-motor AEs.

## Additional file

Additional file 1: **Table S1.** Power calculations. **Table S2.** SNPs included in the study with their predicted and experimentally determined functions. **Table S3.** Excessive daytime sleepiness and sleep attacks, visual hallucinations, and nausea and vomiting. **Table S4.** Orthostatic hypotension, peripheral edema, and impulse control disorders. **Table S5.** Motor fluctuations and dyskinesia. (DOCX 56 kb)

## Abbreviations

AEs: Adverse events
CIs: Confidence intervals
DAs: Dopamine agonists
EDS: Excessive daytime sleepiness
ENS: Enteric nervous system
ICDs: Impulse control disorders
LED: Levodopa equivalent dose
NOS: Nitric oxide synthase
OH: Orthostatic hypotension
ORs: Odds ratios
PD: Parkinson’s disease
PE: Peripheral edema
PET: Positron emission tomography
REM: Rapid eye movement
RNS: Reactive nitrogen species
ROS: Reactive oxygen species
SNpc: Substantia nigra pars compacta
SNPs: Single-nucleotide polymorphisms
VH: Visual hallucinations
