# Supplementary material for: Genetic variability of inflammation and oxidative stress genes does not play a major role in the occurrence of adverse events of dopaminergic treatment in Parkinson’s disease
Source: J Neuroinflammation. 2019 Feb 27;16:50. doi: 10.1186/s12974-019-1439-y (PMC6393982; doi:10.1186/s12974-019-1439-y)
Supplement: Supplementary file 1 — Table S1. Power calculations. Table S2. SNPs included in the study with their predicted and experimentally determined functions. Table S3. Excessive daytime sleepiness and sleep attacks, visual hallucinations, and nausea and vomiting. Table S4. Orthostatic hypotension, peripheral edema, and impulse control disorders. Table S5. Motor fluctuations and dyskinesia. (DOCX 56 kb) [file 12974_2019_1439_MOESM1_ESM.docx]

**Supplementary material:**

**Genetic variability of inflammation and oxidative stress genes does not play a major role in the occurrence of adverse events of dopaminergic treatment in Parkinson's disease**

Sara Redenšek, MPharm^1^, Dušan Flisar, MD^2^, Maja Kojović, MD, PhD^2^, Milica Gregorič Kramberger, MD, PhD^2^, Dejan Georgiev, MD, PhD, FEBN^2^, Zvezdan Pirtošek, MD, PhD^2^, Maja Trošt, MD, PhD^2^, Vita Dolžan, MD, PhD^1*^

^1^Pharmacogenetics Laboratory, Institute of Biochemistry, Faculty of Medicine, University of Ljubljana, Vrazov trg 2, 1000 Ljubljana, Slovenia

^2^Department of Neurology, University Medical Centre Ljubljana, Zaloška cesta 2, 1000 Ljubljana, Slovenia

Additional file 1: Table S1: Power calculations

|  | Motor fluctuations | Dyskinesia | EDS and sleep attacks | Visual hallucinations | Nausea/vomiting | Orthostatic hypotension | Peripheral edema | Impulse control disorders |
| --- | --- | --- | --- | --- | --- | --- | --- | --- |
| min MAF | >3.768 | >3.722 | >3.815 | >4.146 | >3.976 | >3.790 | >4.541 | >5.220 |
| average MAF | <0.371, >2.257 | <0.365, >2.260 | <0.343, >2.326 | <0.293, >2.519 | <0.317, >2.420 | <0.350, >2.307 | <0.243, >2.747 | <0.173, >3.146 |
| max MAF | <0.445, >2.647 | <0.444, >2.691 | <0.432, >2.854 | <0.398, >3.328 | <0.415, >3.080 | <0.435, >2.805 | <0.365, >3.977 | <0.318, >5.471 |

Numbers in the table represent ORs that can be detected with 80% certainty if the difference between groups really exists.

Additional file 1: Table S2: SNPs included in the study with their predicted and experimentally determined functions

| **Gene** | **SNP** | **Location in gene** | **MAF** | **SNP function prediction^a^** | **SNP function**  **[Reference]** |
| --- | --- | --- | --- | --- | --- |
| *NLRP3* | rs35829419 C>A  p.Gln705Lys | Coding region | 0.02 (A) | Nonsynonymous may influence splicing | Gain of function [[1](#_ENREF_1)] |
|  |  |  |  |  |  |
|  |  |  |  |  |  |
| *CARD8* | rs2043211 A>T  p.Phe52Ile | Coding region | 0.32 (T) | May influence splicing Introduces stop codon | A truncated protein is produced [[1](#_ENREF_1)] |
|  |  |  |  |  |  |
|  |  |  |  |  |  |
| *IL1β* | rs16944 A>G  c.-598T>C | 5’UTR | 0.49 (G) | May influence transcription factor binding | Increased production of *IL1β* [[2](#_ENREF_2)] |
|  |  |  |  |  |  |
|  |  |  |  |  |  |
|  | rs1143623 G>C  c.-1560G>C | 5’UTR | 0.29 (C) | May influence transcription factor binding | Decreases promoter activity [[3](#_ENREF_3)] |
|  |  |  |  |  |  |
|  |  |  |  |  |  |
| *TNFα* | rs1800629 G>A  c.-308G>A | 5’UTR | 0.09 (A) | May influence transcription factor binding | A allele carriers have higher protein levels [[4](#_ENREF_4)] |
|  |  |  |  |  |  |
|  |  |  |  |  |  |
| *IL6* | rs1800795 G>C  c.-174G>C | 5’UTR | 0.14 (C) | May influence transcription factor binding | Alters expression [[5](#_ENREF_5)] |
|  |  |  |  |  |  |
|  |  |  |  |  |  |
| *NOS1* | rs2293054 G>A  p.Ile398= | Coding region | 0.25 (A) | May influence splicing | / |
|  |  |  |  |  |  |
|  |  |  |  |  |  |
|  | rs2682826 G>A  c.*276C>T | 3’UTR | 0.26 (A) | May influence miRNA binding | / |
|  |  |  |  |  |  |
|  |  |  |  |  |  |
| *GPX1* | rs1050450 C>T  p.Pro200Leu | Coding region | 0.22 (T) | Nonsynonymous may influence splicing | Decreases enzyme activity [[6](#_ENREF_6)] |
|  |  |  |  |  |  |
|  |  |  |  |  |  |
| *CAT* | rs10836235 C>T  c.66+78C>T | Intron | 0.13 (T) | May influence transcription factor binding | / |
|  |  |  |  |  |  |
|  |  |  |  |  |  |
|  | rs1001179 G>A  c. -262C>T | 5’UTR | 0.13 (A) | May influence transcription factor binding | Increased expression [[7](#_ENREF_7)] |
|  |  |  |  |  |  |
|  |  |  |  |  |  |
| *SOD2* | rs4880 C>T  p.Val16Ala | Coding region | 0.41 (T) | Nonsynonymous may influence splicing | Decreases enzyme activity [[6](#_ENREF_6)] |
|  |  |  |  |  |  |
|  |  |  |  |  |  |

^a^Evaluated using SNP function prediction [[8](#_ENREF_8)].

MAF – minor allele frequency

Additional file 1: Table S3: Excessive daytime sleepiness and sleep attacks, visual hallucinations, and nausea and vomiting

| **Gene**  **SNP** | **Genotype** | **EDS and sleep attacks** | | | **Visual hallucinations** | | | **Nausea and vomiting** | | |
| --- | --- | --- | --- | --- | --- | --- | --- | --- | --- | --- |
|  |  | **OR** | **95% CI** | **p-value** | **OR** | **95% CI** | **p-value** | **OR** | **95% CI** | **p-value** |
| ***NLRP3***  **rs35829419** | CC | Ref. |  |  | Ref. |  |  | Ref. |  |  |
|  | CA | 0.74 | 0.31-1.78 | 0.503 | 0.81 | 0.31-2.11 | 0.662 | 0.81 | 0.32-2.01 | 0.645 |
|  | AA | / |  |  | / |  |  | / |  |  |
|  | CA + AA | 0.70 | 0.30-1.68 | 0.430 | 0.77 | 0.30-2.00 | 0.593 | 0.77 | 0.31-1.91 | 0.570 |
| ***CARD8***  **rs2043211** | AA | Ref. |  |  | Ref. |  |  | Ref. |  |  |
|  | AT | 0.82 | 0.45-1.48 | 0,507 | 0.80 | 0.42-1.53 | 0.498 | 1,312 | 0.71-2.44 | 0.391 |
|  | TT | 0.98 | 0.42-2.27 | 0,962 | 0.79 | 0.31-2.03 | 0.621 | 1,163 | 0.48-2.84 | 0.740 |
|  | AT + TT | 0.86 | 0.50-1.48 | 0.581 | 0.80 | 0.44-1.45 | 0.457 | 1.27 | 0.71-2.27 | 0.414 |
| ***IL1β****  **rs16944** | AA | 1.37 | 0.56-3.35 | 0.490 | 1.06 | 0.38-2.95 | 0.909 | 0.64 | 0.24-1.74 | 0.383 |
|  | AG | 1.21 | 0.67-2.18 | 0.522 | 1.39 | 0.73-2.63 | 0.316 | 0.75 | 0.41-1.39 | 0.357 |
|  | GG | Ref. |  |  | Ref. |  |  | Ref. |  |  |
|  | AA + AG | 1.25 | 0.72-2.16 | 0.435 | 1.31 | 0.72-2.40 | 0.380 | 0.72 | 0.41-1.29 | 0.273 |
| ***IL1β***  **rs1143623** | GG | Ref. |  |  | Ref. |  |  | Ref. |  |  |
|  | GC | 1.15 | 0.64-2.06 | 0.646 | 1,26 | 0.67-2.36 | 0.474 | 0.66 | 0.35-1.25 | 0.204 |
|  | CC | 1.48 | 0.48-4.54 | 0.490 | 0.51 | 0.11-2.40 | 0.395 | 1.16 | 0.37-3.69 | 0.796 |
|  | GC + CC | 1.19 | 0.69-2.08 | 0.532 | 1.13 | 0.61-2.07 | 0.702 | 0.73 | 0.40-1.32 | 0.295 |
| ***TNFα***  **rs1800629** | GG | Ref. |  |  | Ref. |  |  | Ref. |  |  |
|  | GA | 0.76 | 0.40-1.45 | 0.408 | 0.71 | 0.34-1.47 | 0.352 | 0.79 | 0.40-1.55 | 0.484 |
|  | AA | 1.39 | 0.36-5.38 | 0.634 | 1.36 | 0.33-5.67 | 0.676 | 1.13 | 0.27-4.69 | 0.871 |
|  | GA + AA | 0.83 | 0.45-1.52 | 0.547 | 0.78 | 0.40-1.54 | 0.477 | 0.83 | 0.44-1.57 | 0.559 |
| ***IL6***  **rs1800795** | GG | Ref. |  |  | Ref. |  |  | Ref. |  |  |
|  | GC | 0.89 | 0.47-1.66 | 0.708 | 0.73 | 0.38-1.43 | 0.365 | 1.38 | 0.71-2.71 | 0.345 |
|  | CC | 0.96 | 0.42-2.19 | 0.916 | 0.48 | 0.18-1.27 | 0.141 | 0.83 | 0.33-2.10 | 0.693 |
|  | GC + CC | 0.90 | 0.50-1.65 | 0.740 | 0.67 | 0.35-1.27 | 0.218 | 1.23 | 0.64-2.36 | 0.529 |
| ***NOS1***  **rs2293054** | GG | Ref. |  |  | Ref. |  |  | Ref. |  |  |
|  | GA | 1.24 | 0.70-2.21 | 0.465 | 1.10 | 0.59-2.06 | 0.765 | 0.78 | 0.42-1.43 | 0.415 |
|  | AA | 1.87 | 0.67-5.23 | 0.232 | 0.63 | 0.17-2.34 | 0.489 | 1.19 | 0.41-3.47 | 0.745 |
|  | GA + AA | 1.33 | 0.77-2.30 | 0.312 | 1.02 | 0.56-1.86 | 0.960 | 0.84 | 0.50-1.49 | 0.542 |
| ***NOS1***  **rs2682826** | GG | Ref. |  |  | Ref. |  |  | Ref. |  |  |
|  | GA | 1.56 | 0.88-2.79 | 0.130 | 1.14 | 0.60-2.14 | 0.690 | 0.57 | 0.31-1.04 | 0.065 |
|  | AA | **3.73** | **1.22-11.35** | **0.021** | 2.19 | 0.71-6.74 | 0.173 | 0.66 | 0.20-2.22 | 0.502 |
|  | GA + AA | **1.75** | **1.00-3.06** | **0.048** | 1.25 | 0.68-2.29 | 0.471 | 0.58 | 0.32-1.03 | 0.064 |
| ***GPX1***  **rs1050450** | CC | Ref. |  |  | Ref. |  |  | Ref. |  |  |
|  | CT | 1.14 | 0.64-2.03 | 0.649 | **2.01** | **1.07-3.77** | **0.030** | 0.68 | 0.37-1.24 | 0.209 |
|  | TT | 1.06 | 0.37-3.09 | 0.911 | 0.85 | 0.23-3.20 | 0.808 | 0.41 | 0.11-1.52 | 0.183 |
|  | CT + TT | 1.13 | 0.65-1.96 | 0.663 | 1.79 | 0.97-3.31 | 0.061 |  |  |  |
| ***CAT***  **rs10836235** | CC | Ref. |  |  | Ref. |  |  | Ref. |  |  |
|  | CT | 1.16 | 0.60-2.26 | 0.665 | 1.60 | 0.78-3.25 | 0.197 | 1.56 | 0.79-3.10 | 0.205 |
|  | TT | 0.47 | 0.05-4.27 | 0.500 | 2.20 | 0.36-13.63 | 0.397 | 1.77 | 0.29-10.95 | 0.537 |
|  | CT + TT | 1.08 | 0.56-2.05 | 0.827 | 1.65 | 0.84-3.26 | 0.150 | 0.63 | 0.35-1.13 | 0.124 |
| ***CAT***  **rs1001179** | GG | Ref. |  |  | Ref. |  |  | Ref. |  |  |
|  | GA | 1.13 | 0.64-1.98 | 0.674 | 0.67 | 0.35-1.27 | 0.215 | 0.79 | 0.43-1.43 | 0.426 |
|  | AA | 0.20 | 0.03-1.67 | 0.138 | 1.71 | 0.45-6.42 | 0.430 | 0.53 | 0.11-2.60 | 0.430 |
|  | GA + AA | 1.00 | 0.58-1.74 | 0.994 | 0.75 | 0.41-1.37 | 0.345 | 0.76 | 0.42-1.36 | 0.348 |
| ***SOD2***  **rs4880** | CC | Ref. |  |  | Ref. |  |  | Ref. |  |  |
|  | CT | 1.23 | 0.64-2.36 | 0.529 | 1.10 | 0.55-2.21 | 0.788 | 0.53 | 0.28-1.03 | 0.060 |
|  | TT | 1.14 | 0.53-2.48 | 0.735 | 0.69 | 0.28-1.67 | 0.409 | 0.46 | 0.20-1.04 | 0.063 |
|  | CT + TT | 1.20 | 0.65-2.22 | 0.554 | 0.96 | 0.50-1.85 | 0.896 | **0.51** | **0.28-0.94** | **0.030** |

*Recessive model was used.

Additional file 1: Table S4: Orthostatic hypotension, peripheral edema, and impulse control disorders

| **Gene**  **SNP** | **Genotype** | **Orthostatic hypotension** | | | **Peripheral edema** | | | **Impulse control disorders** | | |
| --- | --- | --- | --- | --- | --- | --- | --- | --- | --- | --- |
|  |  | **OR** | **95% CI** | **p-value** | **OR** | **95% CI** | **p-value** | **OR** | **95% CI** | **p-value** |
| ***NLRP3***  **rs35829419** | CC | Ref. |  |  | Ref. |  |  | Ref. |  |  |
|  | CA | 0.80 | 0.34-1.87 | 0.607 | 0.91 | 0.32-2.55 | 0.856 | 0.72 | 0.20-2.53 | 0.604 |
|  | AA | / |  |  | / |  |  | / |  |  |
|  | CA + AA | 0.76 | 0.33-1.76 | 0.520 | 0.87 | 0.31-2.43 | 0.790 | 0.69 | 0.20-2.42 | 0.559 |
| ***CARD8***  **rs2043211** | AA | Ref. |  |  | Ref. |  |  | Ref. |  |  |
|  | AT | 0.98 | 0.55-1.76 | 0.952 | 0.64 | 0.31-1.31 | 0.222 | 0.44 | 0.18-1.07 | 0.069 |
|  | TT | 1.11 | 0.48-2.55 | 0.804 | 0.49 | 0.16-1.53 | 0.217 | 0.90 | 0.30-2.64 | 0.840 |
|  | AT + TT | 1.01 | 0.59-1.74 | 0.961 | 0.60 | 0.31-1.17 | 0.133 | 0.55 | 0.26-1.17 | 0.122 |
| ***IL1β****  **rs16944** | AA | 0.91 | 0.38-2.21 | 0.840 | 0.61 | 0.19-1.93 | 0.399 | 1.54 | 0.45-5.25 | 0.490 |
|  | AG | 0.63 | 0.35-1.13 | 0.121 | 0.60 | 0.29-1.23 | 0.159 | 1.77 | 0.79-3.97 | 0.165 |
|  | GG | Ref. |  |  | Ref. |  |  | Ref. |  |  |
|  | AA + AG | 0.69 | 0.40-1.18 | 0.173 | 0.60 | 0.31-1.17 | 0.133 | 1.72 | 0.80-3.71 | 0.167 |
| ***IL1β***  **rs1143623** | GG | Ref. |  |  | Ref. |  |  | Ref. |  |  |
|  | GC | **0.51** | **0.28-0.93** | **0.028** | 0.60 | 0.29-1.25 | 0.170 | 1.11 | 0.51-2.46 | 0.788 |
|  | CC | 0.99 | 0.33-3.02 | 0.987 | 0.56 | 0.12-2.62 | 0.458 | 1.04 | 0.21-5.02 | 0.964 |
|  | GC + CC | **0.57** | **0.32-1.00** | **0.050** | 0.59 | 0.29-1.19 | 0.140 | 1.10 | 0.52-2.35 | 0.800 |
| ***TNFα***  **rs1800629** | GG | Ref. |  |  | Ref. |  |  | Ref. |  |  |
|  | GA | 0.80 | 0.42-1.51 | 0.494 | 0.81 | 0.37-1.77 | 0.593 | 1.69 | 0.75-3.81 | 0.207 |
|  | AA | 3.29 | 0.79-13.65 | 0.101 | 1.11 | 0.22-5.59 | 0.902 | 2.06 | 0.40-10.65 | 0.389 |
|  | GA + AA | 0.98 | 0.54-1.77 | 0.943 | 0.85 | 0.41-1.76 | 0.655 | 1.74 | 0.80-3.76 | 0.162 |
| ***IL6***  **rs1800795** | GG | Ref. |  |  | Ref. |  |  | Ref. |  |  |
|  | GC | 1.51 | 0.80-2.86 | 0.201 | 0.59 | 0.51-2.29 | 0.831 | 0.95 | 0.41-2.21 | 0.910 |
|  | CC | 0.91 | 0.39-2.14 | 0.829 | 0.90 | 0.19-1.77 | 0.335 | 0.62 | 0.18-2.12 | 0.444 |
|  | GC + CC | 1.34 | 0.73-2.48 | 0.343 | 0.95 | 0.46-1.96 | 0.890 | 0.87 | 0.39-1.95 | 0.731 |
| ***NOS1***  **rs2293054** | GG | Ref. |  |  | Ref. |  |  | Ref. |  |  |
|  | GA | 0.86 | 0.49-1.53 | 0.613 | 1.73 | 0.88-3.41 | 0.111 | 0.85 | 0.38-1.92 | 0.693 |
|  | AA | 0.63 | 0.21-1.90 | 0.412 | 0.31 | 0.04-2.44 | 0.264 | 1.83 | 0.53-6.27 | 0.338 |
|  | GA + AA | 0.82 | 0.48-1.42 | 0.480 | 1.45 | 0.75-2.82 | 0.270 | 0.99 | 0.47-2.10 | 0.979 |
| ***NOS1***  **rs2682826** | GG | Ref. |  |  | Ref. |  |  | Ref. |  |  |
|  | GA | 0.97 | 0.55-1.70 | 0.911 | 1.46 | 0.73-2.90 | 0.283 | 2.02 | 0.91-4.50 | 0.084 |
|  | AA | 1.47 | 0.49-4.35 | 0.491 | 1.24 | 0.32-4.83 | 0.760 | 1.34 | 0.27-6.75 | 0.721 |
|  | GA + AA | 1.02 | 0.60-1.76 | 0.933 | 1.43 | 0.73-2.79 | 0.296 | 1.93 | 0.88-4.22 | 0.100 |
| ***GPX1* rs1050450** | CC | Ref. |  |  | Ref. |  |  | Ref. |  |  |
|  | CT | 0.98 | 0.56-1.72 | 0.935 | 0.87 | 0.44-1.71 | 0.678 | 0.88 | 0.41-1.90 | 0.741 |
|  | TT | 0.66 | 0.22-2.01 | 0.468 | 0.22 | 0.03-1.76 | 0.154 | 0.33 | 0.04-2.67 | 0.301 |
|  | CT + TT | 0.92 | 0.54-1.59 | 0.770 | 0.75 | 0.39-1.46 | 0.400 | 0.79 | 0.37-1.67 | 0.531 |
| ***CAT***  **rs10836235** | CC | Ref. |  |  | Ref. |  |  | Ref. |  |  |
|  | CT | 1.76 | 0.91-3.39 | 0.094 | 0.80 | 0.34-1.85 | 0.596 | 1.58 | 0.67-3.69 | 0.295 |
|  | TT | 2.87 | 0.47-17.67 | 0.255 | / |  |  | / |  |  |
|  | CT + TT | 1.84 | 0.98-3.47 | 0.059 | 0.70 | 0.30-1.63 | 0.410 | 1.39 | 0.60-3.23 | 0.446 |
| ***CAT***  **rs1001179** | GG | Ref. |  |  | Ref. |  |  | Ref. |  |  |
|  | GA | 0.92 | 0.53-1.62 | 0.779 | **0.33** | **0.15-0.70** | **0.004** | 0.55 | 0.24-1.27 | 0.159 |
|  | AA | 0.68 | 0.17-2.74 | 0.582 | 0.30 | 0.04-2.43 | 0.257 | 2.16 | 0.52-9.09 | 0.292 |
|  | GA + AA | 0.90 | 0.52-1.55 | 0.693 | **0.32** | **0.15-0.68** | **0.003** | 0.67 | 0.31-1.45 | 0.314 |
| ***SOD2***  **rs4880** | CC | Ref. |  |  | Ref. |  |  | Ref. |  |  |
|  | CT | 1.31 | 0.69-2.50 | 0.406 | 0.81 | 0.39-1.72 | 0.590 | 0.70 | 0.28-1.71 | 0.429 |
|  | TT | 1.16 | 0.54-2.50 | 0.703 | 0.62 | 0.24-1.60 | 0.324 | 1.34 | 0.51-3.52 | 0.551 |
|  | CT + TT | 1.26 | 0.69-2.31 | 0.450 | 0.75 | 0.37-1.52 | 0.422 | 0.89 | 0.40-2.00 | 0.777 |

*Recessive model was used.

Additional file 1: Table S5: Motor fluctuations and dyskinesia

| **Gene**  **SNP** | **Genotype** | **Motor fluctuations** | | | **Dyskinesia** | | |
| --- | --- | --- | --- | --- | --- | --- | --- |
|  |  | **OR** | **95% CI** | **p-value** | **OR** | **95% CI** | **p-value** |
| ***NLRP3***  **rs35829419** | CC | Ref. |  |  | Ref. |  |  |
|  | CA | 0.68 | 0.30-1.53 | 0.349 | 0.88 | 0.39-1.99 | 0.758 |
|  | AA | / |  |  | / |  |  |
|  | CA + AA | 0.74 | 0.33-1.63 | 0.449 | 0.96 | 0.43-2.14 | 0.919 |
| ***CARD8***  **rs2043211** | AA | Ref. |  |  | Ref. |  |  |
|  | AT | 0.75 | 0.43-1.33 | 0.325 | 1.22 | 0.69-2.17 | 0.488 |
|  | TT | 1.68 | 0.72-3.94 | 0.230 | 1.71 | 0-76-3.88 | 0.196 |
|  | AT + TT | 0.92 | 0.54-1.55 | 0.744 | 1.33 | 0.78-2.23 | 0.288 |
| ***IL1β****  **rs16944** | AA | 1.18 | 0.49-2.84 | 0.706 | 1.24 | 0.52-2.96 | 0.632 |
|  | AG | 1.09 | 0.62-1.91 | 0.766 | 1.05 | 0.60-1.84 | 0.877 |
|  | GG | Ref. |  |  | Ref. |  |  |
|  | AA + AG | 1.11 | 0.66-1.87 | 0.700 | 1.09 | 0.64-1.84 | 0.762 |
| ***IL1β***  **rs1143623** | GG | Ref. |  |  | Ref. |  |  |
|  | GC | 1.11 | 0.63-1.94 | 0.723 | 1.26 | 0.72-2.20 | 0.427 |
|  | CC | 1.24 | 0.41-3.76 | 0.710 | 1.43 | 0.47-4.30 | 0.529 |
|  | GC + CC | 1.13 | 0.66-1.92 | 0.665 | 1.28 | 0.75-2.19 | 0.366 |
| ***TNFα***  **rs1800629** | GG | Ref. |  |  | Ref. |  |  |
|  | GA | 0.93 | 0.51-1.79 | 0.822 | 1.05 | 0.57-1.91 | 0.883 |
|  | AA | 3.16 | 0.64-15.69 | 0.160 | 1.66 | 0.43-6.42 | 0.462 |
|  | GA + AA | 1.08 | 0.61-1.91 | 0.799 | 1.11 | 0.63-1.97 | 0.714 |
| ***IL6***  **rs1800795** | GG | Ref. |  |  | Ref. |  |  |
|  | GC | 0.65 | 0.35-1.20 | 0.164 | 0.84 | 0.46-1.54 | 0.569 |
|  | CC | 0.54 | 0.24-1.20 | 0.128 | 0.69 | 0.31-1.54 | 0.360 |
|  | GC + CC | 0.62 | 0.34-1.11 | 0.108 | 0.80 | 0.45-1.43 | 0.447 |
| ***NOS1***  **rs2293054** | GG | Ref. |  |  | Ref. |  |  |
|  | GA | 1.16 | 0.67-2.01 | 0.603 | 0.75 | 0.43-1.31 | 0.316 |
|  | AA | 0.63 | 0.23-1.77 | 0.384 | 0.60 | 0.21-1.74 | 0.350 |
|  | GA + AA | 1.05 | 0.62-1.78 | 0.854 | 0.73 | 0.43-1.24 | 0.238 |
| ***NOS1***  **rs2682826** | GG | Ref. |  |  | Ref. |  |  |
|  | GA | 0.85 | 0.49-1.46 | 0.550 | 0.79 | 0.46-1.37 | 0.406 |
|  | AA | 1.25 | 0.42-3.74 | 0.695 | 1.02 | 0.34-3.00 | 0.978 |
|  | GA + AA | 0.89 | 0.53-1.51 | 0.663 | 0.82 | 0.48-1.39 | 0.459 |
| ***GPX1***  **rs1050450** | CC | Ref. |  |  | Ref. |  |  |
|  | CT | 1.55 | 0.89-2.70 | 0.120 | 1.23 | 0.71-2.13 | 0.472 |
|  | TT | 1.56 | 0.56-4.38 | 0.400 | 0.98 | 0.35-2.75 | 0.965 |
|  | CT + TT | 1.55 | 0.92-2.63 | 0.103 | 1.18 | 0.70-2.01 | 0.533 |
| ***CAT***  **rs10836235** | CC | Ref. |  |  | Ref. |  |  |
|  | CT | 1.23 | 0.64-2.36 | 0.533 | 1.45 | 0.76-2.77 | 0.261 |
|  | TT | 0.61 | 0.10-3.73 | 0.590 | 0.93 | 0.15-5.68 | 0.934 |
|  | CT + TT | 1.15 | 0.62-2.14 | 0.663 | 1.39 | 0.75-2.59 | 0.301 |
| ***CAT***  **rs1001179** | GG | Ref. |  |  | Ref. |  |  |
|  | GA | 0.89 | 0.52-1.52 | 0.662 | 1.16 | 0.67-2.00 | 0.600 |
|  | AA | 1.27 | 0.34-4.74 | 0.719 | 3.36 | 0.83-13.62 | 0.090 |
|  | GA + AA | 0.92 | 0.54-1.55 | 0.750 | 1.28 | 0.75-2.18 | 0.362 |
| ***SOD2***  **rs4880** | CC | Ref. |  |  | Ref. |  |  |
|  | CT | 1.50 | 0.81-2.79 | 0.200 | 1.23 | 0.66-2.28 | 0.517 |
|  | TT | 0.85 | 0.41-1.77 | 0.658 | 0.72 | 0.34-1.53 | 0.396 |
|  | CT + TT | 1.25 | 0.70-2.22 | 0.456 | 1.04 | 0.58-1.86 | 0.897 |

*Recessive model was used.

**Refernces:**

1. Jenko B, Praprotnik S, Tomsic M, Dolzan V: **NLRP3 and CARD8 Polymorphisms Influence Higher Disease Activity in Rheumatoid Arthritis.** *J Med Biochem* 2016, **35:**319-323.

2. Torres-Merino S, Moreno-Sandoval HN, Thompson-Bonilla MDR, Leon JAO, Gomez-Conde E, Leon-Chavez BA, Martinez-Fong D, Gonzalez-Barrios JA: **Association Between rs3833912/rs16944 SNPs and Risk for Cerebral Palsy in Mexican Children.** *Molecular Neurobiology* 2018, **21:**018-1178.

3. Kutikhin AG, Yuzhalin AE, Volkov AN, Zhivotovskiy AS, Brusina EB: **Correlation between genetic polymorphisms within IL-1B and TLR4 genes and cancer risk in a Russian population: a case-control study.** *Tumour Biol* 2014, **35:**4821-4830.

4. Szkup M, Chelmecka E, Lubkowska A, Owczarek AJ, Grochans E: **The influence of the TNFalpha rs1800629 polymorphism on some inflammatory biomarkers in 45-60-year-old women with metabolic syndrome.** *Aging* 2018, **10:**2935-2943.

5. Lagmay JP, London WB, Gross TG, Termuhlen A, Sullivan N, Axel A, Mundy B, Ranalli M, Canner J, McGrady P, Hall B: **Prognostic significance of interleukin-6 single nucleotide polymorphism genotypes in neuroblastoma: rs1800795 (promoter) and rs8192284 (receptor).** *Clin Cancer Res* 2009, **15:**5234-5239.

6. Esih K, Goricar K, Dolzan V, Rener-Primec Z: **Antioxidant polymorphisms do not influence the risk of epilepsy or its drug resistance after neonatal hypoxic-ischemic brain injury.** *Seizure* 2017, **46:**38-42.

7. Eddaikra A, Amroun H, Raache R, Galleze A, Abdallah-Elhadj N, Azzouz M, Mecabih F, Mechti B, Abbadi MC, Touil-Boukoffa C, Attal N: **Clinical variables and ethnicity may influenced by polymorphism of CAT -262C/T and MnSOD 47C/T antioxidant enzymes in Algerian type1 diabetes without complications.** *Gene* 2018, **670:**182-192.

8. Xu Z, Taylor JA: **SNPinfo: integrating GWAS and candidate gene information into functional SNP selection for genetic association studies.** *Nucleic Acids Res* 2009, **37:**5.
